# ‘Never change a winning team’: GPs’ perspectives on discontinuation of long-term antidepressants

**DOI:** 10.1080/02813432.2021.2006487

**Published:** 2021-12-11

**Authors:** Van Leeuwen Ellen, Sibyl Anthierens, Mieke L. van Driel, An De Sutter, Evelien van den Branden, Thierry Christiaens

**Affiliations:** a Clinical Pharmacology Unit, Department of Basic and Applied Medical Sciences, Ghent University, Ghent, Belgium; b Department of Public Health and Primary Care, Ghent University, Ghent, Belgium; c Family Medicine and Population, Health University of Antwerp, Antwerp, Belgium; d Faculty of Medicine, Primary Care Clinical Unit, University of Queensland, Brisbane, Australia; e Department of Family Medicine and Primary Health Care, Ghent University, Ghent, Belgium; f General practice “Het Spoor”, Antwerp, Belgium

**Keywords:** Mental health, antidepressants, long-term use, discontinuation, qualitative research, general practitioner

## Abstract

**Background:**

Long-term antidepressant use, much longer than recommended by guidelines, can harm patients and generate unnecessary costs. Most antidepressants are prescribed by general practitioners (GPs) but it remains unclear why they do not discontinue long-term use.

**Aim:**

To explore GPs’ views and experiences of discontinuing long-term antidepressants, barriers and facilitators of discontinuation and required support.

**Design and setting:**

Qualitative study in Belgian GPs.

**Method:**

20 semi-structured face-to-face interviews with GPs. Interviews were analysed thematically.

**Results:**

The first theme, ‘Success stories’ describes three strong motivators to discontinue antidepressants: patient health issues, patient requests and a new positive life event. Second, not all GPs consider long-term antidepressant use a ‘problem’ as they perceive antidepressants as effective and safe. GPs’ main concern is the risk of relapse. Third, GPs foresee that discontinuation of antidepressants is not an easy and straightforward process. GPs weigh up whether they have the necessary skills and whether it is worth the effort to start this process.

**Conclusion:**

Discontinuation of long-term antidepressants is a difficult and uncertain process for GPs, especially in the absence of a facilitating life-event or patient demand. The absence of a compelling need for discontinuation and fear of relapse of symptoms in a stable patient are important barriers for GPs when considering discontinuation. In order to increase GPs’ motivation to discontinue long-term antidepressants, more emphasis on the futility of the actual effect and on potential harms related to long-term use is needed.

**KEY POINTS**
Current awareness:
  - Long-term antidepressant use, much longer than recommended by guidelines, can harm patients and generate unnecessary costs.
Main statements:
 • Discontinuation of long-term antidepressants is a difficult and uncertain process for GPs.
 • More emphasis on the futility of the actual effect of antidepressants and on potential harms related to long-term use is needed.

## Introduction

Long-term antidepressant use is rising [1–4]. In the UK, nearly half of the antidepressant users (8% of the total population) have been taking them for more than 2 years [1,2] and in the US two thirds [3,4]. In Belgium, 12.7% of the population take an antidepressant and nearly 30% of those take them for more than 1 year (2018 email from National Institute for Health and Disability Insurance; unreferenced).

Antidepressants that, are not discontinued after the recommended duration, can lead to harm and unnecessary costs. The use of antidepressants puts people at risk of adverse events such as sleep disturbance, weight gain, sexual dysfunction and gastrointestinal bleeding, alongside feeling emotionally numb and addicted [5–9]. Long-term antidepressant use may also impair patients’ autonomy and increase their dependence on medical services and medication [10]. Guidelines for the management of depression recommend that antidepressants should be taken up to 6–12 months after remission and up to 2 years after remission in those at high risk of relapse or with a history of recurrent depression [11,12]. Due to the lack of evidence for the optimal duration of treatment, guidelines are based on expert opinion. There is no recommendation that antidepressants should be taken indefinitely in the primary care population.

Successful discontinuation of antidepressants depends on patient-related factors and doctor-related factors [13]. A review of the patient perspective on discontinuation concluded there is little information about the perspectives of general practitioners (GPs) [13]. This is important as most antidepressants are prescribed by GPs [14,15]. Moreover, patients believe their physician is responsible for initiating discussions about discontinuation [13,16]. Since antidepressants are mostly prescribed by GPs and there are only a few studies exploring their views about stopping the antidepressant, we aimed to explore the perspectives of GPs about discontinuation in long-term antidepressant users.

## Method

### Study design and participants recruitment

We conducted a qualitative study with GPs recruited from two different regions in East-Flanders, Belgium. Purposive sampling was used to ensure the sample represented a wide range of personal characteristics such as sex, age, practice type, and grade of urbanisation. Invitations to participate in the study were sent by email and followed up by phone. Of the 24 contacted GPs, 20 agreed to participate. All GPs provided informed written consent. Participation was voluntary and no compensation was provided.

Ethics approval was obtained from Ghent University, Belgium (reference EC/2019/040).

### Study setting

In Belgium, no gatekeeper function exists and patients are free to consult any GP. However, in recent years patients have been encouraged to enlist with one GP and more than two-thirds of patients have now assigned one GP or group practice to manage their medical file [17]. GP remuneration is mainly based on fee-for-service payment.

In general, GPs refer for psychological treatment to private psychologists and specialised mental health care professionals, however, access is limited due to long waiting lists and high costs. More recently a primary care psychologist has been introduced to improve access to short-term psychological treatment.

### Data collection

A qualitative researcher and GP (EVL) and a GP trainee (EVDB) conducted semi-structured face-to-face interviews in the GP’s practice between May and December 2019 using an interview guide (see Box 1).

Box 1. Interview Guide

Part 1:

- Try to remember at least 1 patient for whom discontinuing long term antidepressant use (>1 year) was successful
  1. **What was your experience and what was the process?**
- Try to remember at least 1 patient for whom discontinuing long term antidepressant use (>1 year) was NOT successful
- **What was your experience and what was the process?**

*[‘Prompts’: why did you discontinue, who started the conversation, how did it go, what worked well, what didn’t work so well, what was the role of other health professionals, psychological and physical capabilities of the patient …]*

Part 2:

1. **Which factors and circumstances make antidepressants (AD) discontinuation possible in your opinion? Why?**
2. **Which factors and circumstances are barriers to AD discontinuation in your opinion? Why?**

*[‘Prompts’: necessary knowledge, skills and attitude are needed, perception of depression and monitoring of AD psychological and physical capabilities of the patient, consequences and impact of discontinuation of AD, what was the role of other health professionals/family/friends, influence of practice related issues, influence of external factors …]*

Part 3:

1. **What support would you need to discontinue AD in more of your patients? Why?**

*[prompts: knowledge and attitude (guidelines/e-learning, training), resources, external factors, role of other health professionals/others]*

The interview guide was based on the literature [18–22]. In brief, participants were asked about their experiences with discontinuation of long-term antidepressant use, barriers, and facilitators on discontinuation and required support. Open-ended questions were followed with prompts to gather further detail. The interviews were audio-recorded, transcribed verbatim, and transcripts were then checked for accuracy and anonymized. The interviews were conducted in the GPs’ practices.

### Data analysis

All transcripts were uploaded in NVIVO v12 and coded thematically by two authors (EVL and EVDB). Three members of the team (EVL, EVDB, SA) independently read five interviews and made notes on key topics and potential themes. The analysis (coding, developing categories and themes) was then discussed in the team and codes and categories were refined and defined in an iterative manner [23]. The key themes were identified, discussed and agreed upon by the full study team.

## Results

### Participants

Demographics of the 20 GPs are provided in Table 1. Twelve GPs were female with ages ranging from 30 to 68 years. The interviews lasted 25 to 60 min.

**Table 1.** Demographics of the 20 FPs.

Age (range in years, years)		Female
25–35	5	3
35–45	4	3
45–55	7	4
55–65	3	2
>65	1	0
Average	44.6	
Sex		
Female	12	
Male	8	
Area		
Rural	10	7
Urban	10	5
Type of practice		
Solo	5	
Duo	3	
Group	10	
Community health centre	2	

### Facilitators and barriers

Three main themes were identified:

- Success stories and the subthemes patient health issues, patient request and positive life event;
- Long-term antidepressants: is there a problem? and the subthemes effective and safe medication, risk of relapse, and routine prescribing;
- Discontinuation of antidepressants is not simple and the subthemes: is it worth the effort?; is it my role?; and ‘GPs need help’.

Table 2 presents quotes illustrating each of the themes.

**Table 2.** Themes and subthemes.

Success stories	
Patient health issues	I recently had someone, a woman, aged 32, who wanted to stop [her antidepressant] because she wanted to get pregnant. Her antidepressant use had started around 8 years ago, I think, in response to a break-up resulting in a depression. That actually went pretty smoothly. I had never considered stopping antidepressant treatment before. She was motivated as well: she said ‘we want a child, it is not possible on AD, it is not allowed, so I am going to stop’. (GP3, female, 58 years)
Patient request	I am thinking of one patient in particular who has been using antidepressants for a very long time, it started with her previous GP. She contacted me of her own accord at a certain point and asked ‘can we stop this treatment doctor?’. I had previously mentioned the possibility on several occasions but had not attempted discontinuation because she dreaded it and thought she had reached a certain psychosocial stability, so I agreed with her. At the point where she reached out, she said ‘I want to try and stop’ and it worked well with a short tapering time of, I think, around a week. (GP1, man, 46 years)
Positive life events	When you think that removal of the tablet is not going to make a difference because they are busy with all sorts of things, e.g. they are happy because of a grandchild, you should do it.(GP8, female, 42 years)
Long-term antidepressant: is there a problem?	
Safe and effective medication	‘they are feeling well now, so it probably has some effect, even if it is only placebo’ (GP18, female, 31 yr) Concerning the side effects, certainly in people who have been taking them for a while, it does not seems to me there are that many. If not much is happening in their life; no new medication, diseases or health issues, there is no incentive to discuss it. So, what is actually the problem? (GP4, man, 32 years)
Risk for relapse	You know, I do not discuss it anymore, what if they relapse? Then it is your responsibility. As doctors we tend to be a little scared of that relapse. (GP6, man, 48 years)
Routine prescribing	‘Yes for those people for whom it is routine medication. In that case you don’t think about it enough or maybe it is more that you don’t think it through… For example, someone on an antihypertensive or with high cholesterol, you know, you are not going to ask questions. Chances of them being cured of that are slim to none. The antidepressant is apparently part of that routine’. (GP14, female, 37 years)
Discontinuation of antidepressants is not simple	
Is it worth the effort?	
Discontinuation is futile/low priority/negative	When you start up medication, you expect a positive result, but with stopping nothing ‘happy’ is going to come from it, it is difficult to stop something, it is like stealing something from your patient. It is still something negative, if you start up medication and people feel better, stopping will not immediately affect the patient, initially it might even have a negative effect. (GP2, female, 35 years)
Discontinuation consumes energy and time	The problem is in the energy consumed by these consults; motivational issues, depression, sleeping issues,… you can’t have too many of them in one day, I don’t know how psychologists manage. This is something we discussed with the receptionist; don’t schedule too many of these consults in one day. It is not an easy group of patients, you’re more likely to discuss something in summer than in winter, there is less energy left over to discuss these things in the middle of flu season as well. (GP8, female, 42 years)
Is it my role?	
Patients do not ask to discontinue the medication	When people have been taking antidepressant for years it is very difficult, they will say ‘doctor you have asked that already and I told you I am not experiencing side effects, I want to keep taking it’. Sometimes they even get a bit annoyed. Six months later you are asking the same question. That is hard. They might think you are messing with them. If you keep running into a brick wall you will give up at a certain point. (GP10, man, 65 years)
Locus of control with other clinician	Well, no, a lack of trust is not the right way to put it but the other doctor told them to take that medication. At that moment they have a consultation for something else that they trust you with but changing that other tablet will need to be tackled by the one who initiated the treatment. (GP15, man, 29 years)
‘GPs need help’	
Collaborative management	If psychologists would indicate that patients have been stable for a long time that would help as well. If they would say: ‘I do not see any alarming signs anymore, they told me the following things had happened and they responded in this way, so I notice that there is an improved coping mechanism right now’. If you would get that kind of feedback you would feel more confident that it will work. (GP2, female, 35 years)
Information skills and tools	If I could print a leaflet and give it to the patient with some extra information, that would really help me. Right now you take away their antidepressant and send them on their way with nothing. Being able to give them something to take home would help. (GP20, female, 32 years)

### Theme 1 success stories

Overall, in GPs experiences withdrawal symptoms are acceptable if you inform the patient about withdrawal symptoms at initiation and cessation of the antidepressant and the discontinuation is done gradually. Three particular situations are strong motivators for our participants to discontinue antidepressants: patient health issues, patient requests and new positive life event. They considered the chance of success to be very high in these situations.

#### Patient health issues

All GPs discontinued antidepressants in response to a patient health issue, such as side effects, a pregnancy, or a contra-indication for antidepressants due to new diseases/medication. They pointed out that such a clear patient health issue usually triggers them or the patient to raise the topic. Given the risk of harm associated with continuation, patients were in those cases more motivated to stop the antidepressant.

#### Patient request

All GPs agreed to stop the long-term antidepressant and guide the patient through the discontinuation process when this was requested by the patient. They considered the patient’s motivation as an important marker for readiness and the right condition to discontinue. A patient request was seen as an opportunity to discontinue. This is because this means the GP did not have to raise the subject himself or face difficult discussions to overcome the patient’s resistance. However, they mentioned that such a patient request was uncommon.

#### Positive life event

Several GPs stated that the optimal conditions for discontinuation were essential. However, the patient being psychologically stable was not considered as a strong motive for discontinuation in its own right. Our GPs described they wait for ‘the optimal moment‘. They explained that they only attempt discontinuation if an ‘alternative’ to the antidepressant is available. For instance, a new positive life event or lifestyle such as a new hobby, new (voluntary) work, new grandchild, a new relationship may function as an alternative and facilitate discontinuation. One GP explained: *‘When you think that removal of the tablet is not going to make a difference because they are busy with all sorts of things, e.g. they are happy because of a grandchild, you should do it’.* (GP8)

### Theme 2 long-term antidepressants: is there a problem?

Long-term use of antidepressants was not perceived as a ‘problem’ by GPs: antidepressant are effective and safe and discontinuation could cause unnecessary problems. They prefer routine prescribing with a well patient.

#### Effective and safe medication

Antidepressants were regarded as effective, even in cases where GPs attributed their effectiveness solely to the placebo effect. GPs considered that the antidepressant worked well and helped patients remain stable even after many years. As one GP commented: *‘… … whether the effect is actually realistic or not is irrelevant, for 10 years you’ve experienced a positive feeling from it, that will not go away’.* (GP6)

Some GPs believed that antidepressants help correct the chemical imbalance in the brain caused by the depletion of serotonin in people with depression and are necessary to ensure the psychological stability of the patient’s condition even after several years.

GPs considered antidepressants as safe and well-tolerated by patients. They saw few side effects from long-term use and people did not report them. They assumed that antidepressants would have been stopped at the start if people were experiencing side effects.

Several GPs asked patients on long-term antidepressants to make a review appointment at least once a year to discuss their well-being and to confirm their remission. However, this was not a review to consider deprescribing: GPs saw the patient’s remission as confirmation that the antidepressants were effective and necessary. As a result, they continued the antidepressant if the patient was feeling well. They did not even consider discontinuation.

#### The risk of relapse

GPs’ main concern about discontinuation was the risk of destabilizing a patient who was feeling well. They would feel responsible for a possible relapse and are therefore trying to avoid it. They were also afraid patients would blame them if they relapsed after discontinuation, and which would also make GPs feel guilty.

The unpredictable risks of discontinuation were used to justify continuation. One GP explained*: ‘I think it is like the phrase “Never change a winning team”, everything is fine’.* (GP3)

Some GPs were so concerned about the risks associated with discontinuation that they never consider discontinuation in difficult situations, even if their patient asks. All GPs reported they were less likely to discontinue the antidepressant in case of ongoing adverse life circumstances, such as relationship problems, financial worries, work-related issues, behavioural problems of children, renovations, struggles with stress, especially if patients were unable to change these circumstances. Some GPs also argued that by continuing the antidepressant they show empathy with the patient’s problems. As one GP said: *‘when using your empathic abilities, you can imagine them needing antidepressants to get through the day’.* (GP6)

GPs considered antidepressants the best option for coping with the situation and ceasing the antidepressant as simply ‘not done’. Many GPs were not inclined to discuss the issue in autumn/winter or around Christmas time or other holidays. They assumed people would not feel well or be lonely, increasing the risk of destabilization.

Several GPs acknowledged that they hesitate to discuss the reason for initiation of the antidepressant when evaluating how things have changed in their patient’s life, even with a well-known patient where they initiated the antidepressant themselves. They did not want to confront the patient with a difficult episode of their past, and were afraid that reliving the miserable period could destabilise a currently well patient: *‘that chapter of the person’s life is closed, I do not want to bring that up again while they are not experiencing any side effects, they are feeling fine, so, you know, let’s just give it a rest’.* (GP19)

#### Routine prescribing

Routine prescribing after the acute depressive episode was very common. The usefulness of a routine prescription is not really questioned. Continuation maintained the status quo: *‘it has become a routine and a convenience. It is really a convenience for both parties’.* (GP14)

Most GPs allowed patients to request a repeat prescription without an appointment. It seems that GPs preferred to prescribe without an appointment as mostly no ‘meaningful’ review happens during the consultation. They explained that when the antidepressant prescription is part of their routine for bulk-prescribing long-term repeat prescriptions, a review of the need for this repeat is missing.

GPs found changing their routines and implementing a regular proactive review of the antidepressant difficult. This entails abandoning the easy path of maintaining the status quo. Indeed, some GPs describe themselves as ‘reactive’ and do not review proactively.

### Theme 3 discontinuation of antidepressants is not simple

When GPs reflect on the inappropriateness of the antidepressant and consider discontinuation, they encounter barriers on different levels.

#### Is it worth the effort?

GPs expressed very little enthusiasm about the discontinuation of antidepressants: they see it as a ‘difficult job’. They dread the energy and time-demanding process. Given their perception that discontinuation is futile and negative, they doubt if discontinuation is worth the effort.

##### Discontinuation is futile/low priority/negative

Even GPs who are aware of inappropriate antidepressant use, perceived discontinuation as a lower priority. They frequently reported time constraints on the consultation, however, they were not inclined to ask the patient to make a new appointment for a review. As one GP mentioned: *‘I, only ask someone to come back if we think it is medically necessary or if it is very clear; e.g. with an infection, is it going well, do we need to start antibiotics or not?’ (GP5)*

This view on discontinuation was echoed by many GPs who framed discontinuing antidepressants negatively in their communication. Most see discontinuation as ‘giving bad news’ and described discontinuation as ‘taking something away from the patient’. None of the GPs framed discontinuation antidepressants positively or emphasised that it is a positive message that carries benefits for the patient (e.g. final recovery from depression, enhanced self-confidence of the patient). They did not express any approaches to overcome this negative framing.

GPs assumed that patients expect medications from their doctor and may perceive discontinuation or ‘doing nothing’ as a choice of lower value. GPs explained that ‘feeling the same’ as before discontinuation is not perceived as a positive consequence of discontinuation by the patient. As a GP said: *‘When you start up medication, you expect a positive result and people feel better, but with quitting nothing “happy” is going to come from it, … stopping will not immediately affect the patient’.* (GP2)

##### Discontinuation consumes energy and time

Most GPs described the process of antidepressant discontinuation as difficult and complex and requires a lot of energy and time compared to prescribing. One GP argued: *‘If they are doing well on their current pills, why should I start messing around with it? What if everyone who comes here, stays for a 45 min talk? It needs to stay manageable’.* (GP3)

Moreover, some GPs found it difficult to discuss psychological problems in general. For example, one GP argued: *‘The problem is in the energy consumed by these consults; motivational issues, depression, sleeping issues,… you can’t have too many of them in one day’.* (GP8)

Many tended to raise the issue in spring and summer instead of winter due to the lower workload in their practice and is in line with the result that they are not inclined to discuss the issue in winter due to the higher risk of destabilisation.

#### Is it my role?

Some GPs question if it is their role to raise the issue. They seem to prefer the patient or even other doctors to take this responsibility.

##### Patients do not ask to discontinue the medication

GPs said that when a patient did not ask or express a desire to discontinue there was no reason to raise the issue. They assumed that if patients don’t ask to stop, they continue the antidepressant. Others see it as the patient’s responsibility to actively ask to discontinue the medication and the GP’s duty to provide support for discontinuation.

Nearly all GPs were concerned about jeopardising a (good) patient relationship by raising the topic when a patient did not ask to discontinue. Others did not bring up the issue to avoid conflicts with their patient or lose patients to other doctors. Therefore, some found it easier to discuss the topic with new patients rather than with a patient they are familiar with. However, all GPs emphasised that knowledge of the initial depressive disorder, the triggers, and the reason why antidepressants were initiated are important when considering discontinuing.

##### Locus of control with other clinicians

Nearly all GPs reported they ‘inherited’ patients with long-term use initiated by other doctors. As they assume that a thoughtful decision to continue treatment had been made, they do not question a prescription request from these patients, rather they put the responsibility for discontinuation on the treating colleague or on the initial prescriber, who may have neglected to do this. They struggled also with the patient's loyalty to the previous doctor. By ascribing the responsibility for discontinuation to other doctors, GPs reduced their own responsibility in the discontinuation process. However, usually, they would not make any effort to contact the previous doctor to obtain more information. Further, some GPs described they were more uncomfortable to question a psychiatrist’s prescribing decisions due to the (assumed) more severe degree of the condition for which antidepressants were prescribed and out of respect for a perceived ‘medical hierarchy’.

On the other hand, all GPs thought it was easier to raise the issue if they had initiated the antidepressant themselves and had explained that discontinuation is going to be needed.

#### ‘GPs need help’

Some GPs felt they had to face the difficult task of discontinuation on their own and said additional help was useful.

##### Collaborative management

Some preferred collaboration with psychologists (or social workers or nurse practitioners) to provide additional support but this was not common practice. They saw a potential role for psychotherapists in improving the patient’s self-efficacy and managing their fears. Some stated they want more teamwork to share the responsibility of managing discontinuation. GPs acknowledged that the availability of accessible psychotherapy is limited and noticed (or assumed) that not all patients are motivated to follow psychotherapy. However, they emphasised that the current collaboration and communication between GPs and psychotherapists is rather poor. Some mentioned they doubted if psychologists have sufficient expertise and experience to support the discontinuation process.

##### Information, skills and tools

Most GPs felt confident in their knowledge and skills to guide a patient during the discontinuation process. Nevertheless, several mentioned that guidelines provide no clear information on managing long-term antidepressants and suggested a practical step-by-step guide to discontinue long-term use. Others thought that an information leaflet for patients may be useful.

Other GPs reported a gap in communication skills to overcome a patient's resistance or to bring it up after many years of continuation. Audit and feedback were suggested to give insight into their prescribing. However, they do not really know how they can change their current prescribing. Some GPs thought that an electronic warning to alert the end of treatment could be useful.

## Discussion

### Main findings

This study is one of few studies providing insights into the perceptions of GPs when it comes to (dis)continuation of antidepressants and suggests that GPs are not inclined to discontinue antidepressants proactively. Multiple barriers were identified. Long-term use of antidepressants was not seen as a ‘problem’ by GPs because they perceived the treatment as useful and safe. Their main concern was risk of relapse. When considering discontinuation, they are hesitant and doubt if depriving the patient of the antidepressant is worth the effort. We found mixed opinions regarding the responsibility to raise the topic. GPs do not know how to start discontinuation and want more support. Additionally, we found that GPs hesitate to bring up a miserable period from the past and routine repeat prescribing was common.

### Strengths and limitations

We purposely sampled 20 GPs from 2 different geographic regions in Belgium to ensure we fully captured the breadth of opinions and experiences and the data reached saturation. Some data are specific to the Belgian context (in particular collaboration with a psychologist) however most themes are generalisable to other primary care settings. Because we focused on adults in the community, our results may not be transferable to older people in nursing homes and younger people.

### Comparison with existing literature

Studies have shown that patients regard their GP as the person responsible for initiating discussions about stopping long-term antidepressants [13]. However, our study indicates that GPs are not inclined to do this. Previous studies [18,20,24] confirm this finding even when GPs consider it is their responsibility to raise the issue or see it as a shared decision. Moreover, some of our GPs openly question if it is their role to raise the issue and expect patients to contact them when the patient wants to discontinue, which is in line with Bosman’s findings [18]. As a result, patients and GPs are each waiting for the other to raise the issue of discontinuation, and meanwhile, the status quo is maintained. A recent study showed this lack of initiative and support from the doctor can result in a cyclic process of patients stopping and restarting on their own and/or leading to help-seeking outside mainstream health care (such as online fora) [25,26]. This suggests that GPs should take the lead to frequently review the treatment. Making stopping agreements in advance and maximizing shared decision-making seems important. However, a recent study highlighted the relational and social setting of discontinuation. GPs want to make sure their patient is in a stable situation and ready for a discussion about discontinuation before they raise the issue. Therefore, initiating a discussion with a patient is a complex process [24].

Many of our GPs do not seem to be concerned about the risks associated with the long-term use of antidepressants which is confirmed in other studies with GPs and patients [13–14]. It seems these beliefs, are based on their experience and not consistent with studies showing that antidepressants may cause many adverse events [5–10]. They were much more concerned about the risk of harm of discontinuation, which is in line with other studies [14,18,20,22,24]. The risk of relapse during and after discontinuation is realistic but seems to be lower than estimated by our GPs. An important group of people on long-term antidepressants can stop the antidepressant [27]. There is also evidence that when supported by psychotherapy, 40–75% of people with recurrent depression are able to discontinue their antidepressants [28–30].

Somewhat surprising was that GPs say that by continuing the antidepressant in patients with difficult life circumstances they are showing empathy. This may be explained by their desire to help their patient. Nonetheless, a non-pharmacological approach such as listening, psycho-education, or a referral to the appropriate service would seem more appropriate. This finding is in line with Johnson who found that medicalisation of appropriate misery due to life events plays an important role in the initiation of an antidepressant [14], this seems to also play a role in the process of (dis)continuation.

Another surprising finding was that many of our GPs framed discontinuation of long-term antidepressants negatively and to our knowledge, this is the first time this is reported so extensively. Our GPs perceive discontinuation as bringing bad news for the patient. Indeed, a positive attitude of the prescriber towards discontinuation and a belief that discontinuation can be beneficial are known facilitators for the discontinuation process [31–32].

Some of our GPs also wanted more collaboration with other health care professionals [18–22,24]^.^ Bosman et al. [18] suggest that mental health assistants could provide support, for example, by monitoring antidepressant use and providing supportive guidance during discontinuation. Bowers et al. [20] identified a role for care coordinators. Evidence suggests that a collaborative care model can improve depression outcomes and AD adherence. However, the effect on successful discontinuation has not been evaluated [33–34]. Moreover, qualitative studies found that GPs express doubts about the value of depression care managers including concerns about role overlap [35,36] or about nurses’ authority and experience in managing antidepressants [20]. Overall, a collaborative care model can be useful in improving discontinuation, but further research is needed to explore the views and roles of other professionals involved. Indeed, a Dutch study found that in 5 years following the introduction of mental health nurses in GP practices, the number of AD prescriptions has not been decreased [37].

### Implications for practice and policy

These findings have significant implications for interventions to improve the discontinuation of antidepressants in clinical practice. A step-by-step approach is necessary. First, more emphasis is needed on the potential risks of long-term use and the lack of evidence for a long-term effect. But second, risks must be reframed focusing on the benefits of discontinuation and a realistic view on the risks of discontinuation. Third, patients need to be empowered to understand the risks and benefits of long-term use Trusted health evidence can help people prepare for the consultation and make an informed shared decision [38].

Further research needs to investigate how to support those GPs including support from multidisciplinary care and provide advice on the most appropriate and effective withdrawal strategies.

Education for GPs about the discontinuation of long-term antidepressants should take into account the findings of our study. Changing prescribing behaviour is a well-known challenge and interventions should be tailored to the individual GP’s capability, motivation, and opportunity as described in the Behaviour Change Wheel [39]. For instance, if knowledge about the long-term effects of antidepressants is a gap, capability in terms of knowledge needs to be addressed. However, motivation refers to habits that drive behaviour and need a different approach. Antidepressant prescribing is often a ‘routine’ and breaking the inertia of routines is difficult. Sharing success stories may inspire GPs and help tackle the inertia.

## Conclusions

Discontinuation of long-term antidepressants is a difficult and uncertain process for GPs, certainly in the absence of a facilitating life event or patient demand. GPs’ continued antidepressant prescribing can be seen as a form of empathizing with their patients. The fear of destabilizing a currently well patient is an important barrier for GPs when considering discontinuation. GPs are unaware of the futility of antidepressant continuation and the negative aspects of long-term use. More education, guidance, and support for GPs and patients are needed to reduce unnecessary treatment.
